# MicroRNA as a promising molecular biomarker in the diagnosis of breast cancer

**DOI:** 10.3389/fmolb.2024.1337706

**Published:** 2024-05-15

**Authors:** Felipe Silva de Miranda, José Slaibi-Filho, Gabriel Calasans dos Santos, Nathalia Teixeira Carmo, Carla Martins Kaneto, Thaiz Ferraz Borin, Wilson Barros Luiz, Luciene Cristina Gastalho Campos

**Affiliations:** ^1^ Department of Biological Science, State University of Santa Cruz, Ilhéus, Bahia, Brazil; ^2^ Laboratory of Applied Pathology and Genetics, State University of Santa Cruz, Ilhéus, Bahia, Brazil; ^3^ Department of Health Sciences, State University of Santa Cruz, Ilhéus, Bahia, Brazil; ^4^ Laboratory of Tumor Angiogenesis, Georgia Cancer Center, Department of Biochemistry and Molecular Biology, Augusta University, Augusta, GA, United States

**Keywords:** liquid biopsy, cancer diagnosis, circulating miRNA, oncology, personalized medicine

## Abstract

**Introduction:** Breast cancer represents the most prevalent malignancy among women. Recent advancements in translational research have focused on the identification of novel biomarkers capable of providing valuable insights into patient outcomes. Furthermore, comprehensive investigations aimed at discovering novel miRNAs, unraveling their biological functions, and deciphering their target genes have significantly contributed to our understanding of the roles miRNAs play in tumorigenesis. Consequently, these investigations have facilitated the way for the development of miRNA-based approaches for breast cancer prognosis, diagnosis, and treatment. However, conducting a more extensive array of studies, particularly among diverse ethnic groups, is imperative to expand the scope of research and validate the significance of miRNAs. This study aimed to assess the expression patterns of circulating miRNAs in plasma as a prospective biomarker for breast cancer patients within a population primarily consisting of individuals from Black, Indigenous, and People of Color (BIPOC) communities.

**Methods:** We evaluated 49 patients with breast cancer compared to 44 healthy women.

**Results and discussion:** All miRNAs analyzed in the plasma of patients with breast cancer were downregulated. ROC curve analysis of miR-21 (AUC = 0.798, 95% CI: 0.682–0.914, *p* <0.0001), miR-1 (AUC = 0.742, 95% CI: 0.576–0.909, *p* = 0.004), miR-16 (AUC = 0.721, 95% CI: 0.581–0.861, *p* = 0.002) and miR-195 (AUC = 0.672, 95% CI: 0.553–0.792, *p* = 0.004) showed better diagnostic accuracy in discrimination of breast cancer patients in comparison with healthy women. miR-210, miR-21 showed the highest specificities values (97.3%, 94.1%, respectively). Following, miR-10b and miR-195 showed the highest sensitivity values (89.3%, and 77.8%, respectively). The panel with a combination of four miRNAs (miR-195 + miR-210 + miR-21 + miR-16) had an AUC of 0.898 (0.765–0.970), a sensitivity of 71.4%, and a specificity of 100.0%. Collectively, our results highlight the miRNA combination in panels drastically improves the results and showed high accuracy for the diagnosis of breast cancer displaying good sensitivity and specificity.

## 1 Introduction

Breast cancer is the most common type of cancer in the world among women, excluding non-melanoma skin cancer, and has surpassed lung cancer with an estimated 2.3 million new cases per year with almost 700,000 deaths (Sung et al., 2021). It is a heterogeneous disease comprising multiple biological subtypes, with different clinical patterns lack of intertumoral and intratumoral uniformity, and with wide variation in tumors that imply specific prognoses and treatments (Waks and Winer, 2019). There is a strong genetic component in the pathophysiology of cancer and this point can be explored in clinical practice through molecular biomarkers such as cfDNA and miRNA. The expression level of several miRNAs is closely linked to morphological characteristics, immunohistochemical profiles, histopathological parameters, clinical outcomes, prognosis, and responses to breast cancer treatment (Ohzawa et al., 2017; Dastmalchi et al., 2020).

The initial evidence of miRNA involvement in cancer was described by Calin et al. (2002). In 2008, Lawrie et al. identified them for the first time as potential cancer biomarkers in patients with diffuse large B-cell lymphoma (Lawrie et al., 2008). Since the role of miRNAs regulation in cancer was first reported, several studies have shown altered expression of miRNAs in breast cancer. Comparative methods of miRNAs expression in normal and neoplastic tissues began to be used, intending to establish expression profiles of these that could help in the diagnosis, classification, and prognosis of various types of cancer, including the profiles in different subtypes of some tumors (Dastmalchi et al., 2020; Santana et al., 2023).

Research on circulating miRNAs in oncology has primarily focused on their utilization as diagnostic biomarkers for monitoring asymptomatic high-risk individuals and identifying cancer patients. They have also been explored as prognostic biomarkers, predicting clinical outcomes and monitoring disease recurrence, as well as predictive biomarkers for monitoring therapeutic response and sensitivity to therapy, though all with varied results (Condrat et al., 2020; Jordan-Alejandre et al., 2023). Currently, there is no commercially available panel of miRNAs. Existing molecular marker panels in oncology, such as MammaPrint, Oncotype Dx, and PAM50, are based on identifying genetic changes in specific genes. These panels exhibit varying levels of accuracy and can detect molecular changes before clinical symptoms appear (Xin et al., 2017). However, there are 55 studies registered on the Clinical Trials platform that involve miRNA and breast cancer, utilizing it as a diagnostic or prognostic biomarker or as an auxiliary tool in therapy monitoring (NIH, 2023).

Thus, miRNAs have begun to attract considerable interest for their regulatory involvement in breast cancer initiation, progression, and metastasis. Lastly, it is crucial to highlight that miRNAs can be easily detected in tumor biopsies and are also consistently present in body fluids, especially in blood, plasma, serum, and saliva. They are protected from RNAse activity by being associated with HDL or Ago2, or enclosed in exosomes, making them stable and allowing for their utilization as minimally invasive diagnostic tools in liquid biopsy (Ashby et al., 2014; Bahrami et al., 2018; Li et al., 2021).

Therefore, the aim of this study is to evaluate the expression of circulating miRNAs (miR-1, miR-10b, miR-16, miR21, miR-34a, miR-195, and miR-210) in plasma as a potential biomarker for breast cancer patients. The association of these circulating miRNAs with molecular subtypes and with expression levels in healthy patients was also analyzed. Finally, we further investigated the predictive models of target genes and biological pathways of these circulating miRNAs.

## 2 Materials and methods

### 2.1 Patients and clinical samples

The study was developed using a prospective, cross-sectional, and quantitative approach. A non-probability sampling by convenience was performed. Breast cancer patients and controls were all selected from an ambulatory oncology care unit in the southern region of Bahia state, Brazil from October 2019 to October 2020. This ambulatory unit was chosen because it is a regional clinical reference center in oncology and treats patients through the Brazilian Public Unified Health System (SUS) and private health insurance. Written informed consent was provided to all patients and healthy controls. This study was approved by the Committee for Ethics in Research of the State University of Santa Cruz—UESC (CAAE 19466419.4 .0000.5526), in accordance with Brazilian human research legislation of National Health Council (CNS) Resolution 466/2012 (Brazil, 2012) and the Declaration of Helsinki (UN, 1965).

We selected eligible breast cancer patients based on the following criteria: female patients, over 18 years old, with a histopathological diagnosis of breast cancer and the molecular subtype defined by immunohistochemistry test. Exclusion criteria were considered as a patient with mesenchymal neoplasm, or with metastatic disease at the time of diagnosis, or previous report of any other type of cancer. During the medical consultation, a structured questionnaire was applied to identify the sociodemographic profile, risk factors, and individual clinical-pathological aspects. Then, the patients were referred for venous blood collection in the institution’s laboratory, promptly after the medical consultation diagnosing and before being submitted to any surgical, chemotherapy, or radiotherapy treatment.

For the healthy control group, we selected women considered healthy after clinical and imaging evaluation demonstrating the absence of malignancy, absence of benign tumors, in addition to having no personal history of neoplasia and family history of breast/ovarian cancer. The healthy control group is age-matched with the breast cancer group. There were no significant differences in age between the groups (*p* = 0.236).

Plasma sample from patients and controls was obtained using the following collection and sampling procedures: 4 mL peripheral blood was sampled in BD Vacutainer tubes with EDTA (BD, New Jersey, United States), centrifuged at 2000 *g* for 10 min at room temperature. The supernatant was transferred to a microcentrifuge tube and stored at −80°C, until RNA extraction.

### 2.2 Plasma preparation for RNA isolation

For the extraction of total RNA from plasma, 400 µL of plasma was used. TRIzol reagent (Invitrogen, Massachusetts, United States) was added, following the manufacturer’s recommendations with some modifications. Briefly, a 2.5:1 TRIzol:sample ratio was used. After the addition of TRIzol, the mixture was vortexed for 1 min followed by incubation for 10 min at room temperature. Chloroform P.A. (Synth, Sao Paulo, Brazil) was added in a 5:1 TRIzol:chloroform ratio, each tube was shaken by inversion and incubated for 10 min at room temperature. To complete the separation, the samples were centrifuged for 15 min, 12,000 g at 4°C. After centrifugation, 500 µL of the aqueous phase was transferred to a new microtube. For RNA precipitation, isopropanol P.A. (Neon, Sao Paulo, Brazil) and 1 µL of glycogen (5 mg/mL—Invitrogen, Massachusetts, United States) were added in a 2:1 TRIzol:Isopropanol ratio. The mixture was incubated at −20°C for 16 h. The pellet was visible at the bottom of the microtube after centrifugation for 30 min with 16,000 g and a temperature of 4°C. To eliminate phenol and salt residues, the total RNA in the sample was washed with 75% ethanol (Honeywell, North Carolina, United States), homogenized, and centrifuged for 10 min at 12,000 g and 4°C. Then, the RNA pellet was air-dried for 20 min for further solubilization in DEPC-treated water for RT-PCR (Invitrogen, Massachusetts, United States) and stored at −20°C for subsequent analysis.

The concentration and quality of total RNA were analyzed by NanoDrop ND-2000 spectrophotometer (Thermo Fisher Scientific, Massachusetts, United States). 700 ng of total RNA was used for cDNA synthesis, using specific primers for each miRNA studied and the TaqMan MicroRNA Reverse Transcription Kit (Applied Biosystems, Massachusetts, United States), using a final volume of 15 µL for the reaction, according to the manufacturer’s instructions. The conditions for cDNA synthesis followed three sequential incubations: (i) 16°C for 30 min, (ii) 42°C for 30 min, and (iii) 85°C for 5 min. After synthesis, the cDNA was diluted in DEPC-treated water to the final volume of 50 µL and stored at −20°C for subsequent analysis.

### 2.3 Quantitative reverse transcription polymerase chain reaction (RT-qPCR)

The individual expression levels of miRNAs were detected by the RT-qPCR technique using the QuantStudio 3 equipment (Thermo Fisher Scientific, Massachusetts, United States) and the TaqMan Fast Advanced Master Mix assay (Thermo Fisher Scientific, Massachusetts, United States), under standard cycling conditions, according to the manufacturer’s recommendations. The amplification reaction consisted of 50 ng of cDNA, TaqMan Master Mix, and a specific probe for each miRNA: hsa-miR-1-3p (ID 002222, miRbase MI0000651) (Liu et al., 2017; Wu, 2018; Peng et al., 2020; Chen et al., 2021); hsa-miR-16-5p (ID 000391, miRbase MI0000070) (Qu et al., 2017; Tommasi et al., 2021; Wang et al., 2021); hsa-miR-21-5p (ID 000397, miRbase MI0000077) (Wu et al., 2012; Li et al., 2016; Chen et al., 2019; Wang et al., 2019); hsa-miR-210-3p (ID 000512, miRbase MI0000286) (Evangelista et al., 2021; Khalilian et al., 2023; Santana et al., 2023); hsa-miR-195-5p (ID 000494, miRbase MI0000489) (Singh et al., 2015; McAnena et al., 2019; Chen et al., 2022); hsa-miR-10b-5p (ID 002218, miRbase MI0000267) (Han et al., 2014; Nassar et al., 2014; Zhang et al., 2018; Raval et al., 2022), and hsa-miR-34a-5p (ID 000426, MI0000268) (Peurala et al., 2011; Misso et al., 2014; Zaleski et al., 2018; Chen et al., 2021). Assay ID for TaqMan MicroRNA Assay, Thermo Fisher Scientific.

The qPCR conditions were: incubation for 3 min at 95°C followed by 40 cycles of 15 s at 95°C and 1 min at 60°C. Ct values for RT-qPCR were determined using QuantStudio Design and Analysis Software (Applied Biosystems, Massachusetts, United States) (RRID:SCR_020238). PCR reactions were performed in duplicate and experiments with coefficients of variation greater than 5% or that exhibited unusual amplification curves were excluded. An assay control, without cDNA (NTC), was included as described above. The average of the Ct values of the duplicates was used to calculate the target miRNA expression. For normalization, has-miR-222-5p (Assay ID 002097—ThermoFisher Scientific, Massachusetts, United States) was used as an endogenous control. Target miRNA expression was calculated, first, using the ΔCt formula, which represents the difference between the mean obtained for the transcript of interest and the mean calculated for the endogenous normalizer:

$$∆Ct=Ct\;\left(target\;miRNA\right)-Ct\;\left(\text{endogenous normalizer}\right)$$

Then, the ΔΔCt formula was applied, considering the control group sample as a calibrator for the case group, assigning the zero value to the calibrator as a result of the difference between the values of its ΔCt, for the calculation of differential expression:

$$∆∆Ct=∆Ct\;\left(target\;sample\right)-∆Ct\;\left(mean\;ofcontrol\;group\right)$$

Lastly, the scientific notation formula was applied (Livak and Schmittgen, 2001):

$$2^{-∆∆Ct}$$

### 2.4 miRNA selection for endogenous normalizer

An initial screening was performed to select the miRNA with stable intra-sample and inter-group expression for application as an endogenous normalize in this study, between samples from breast cancer patients and healthy controls. Using two open-source software: Reffinder (RRID:SCR_000472) and NormFinder (RRID:SCR_003387), four miRNA candidates for endogenous normalizers (has-miR-1, has-miR-10 b, has-miR-16, and has-miR-222) were tested (Xie et al., 2012; Prado et al., 2019). The assay was carried out as described above. Then, the generated data were entered into two platforms: (i) Reffinder online tool (https://www.heartcure.com.au/reffinder/) composed of four different normalization tools (Bestkeeper, NormFinder, Genorm, Comparative Delta-Ct method) that use different algorithms to evaluate gene expression more stably and (ii) NormFinder application, an additional extension module for Microsoft Excel 2019 (Microsoft Corporation, New Mexico, United States), that ranks the set of candidate normalization genes according to their expression stability in a given sample set and given experimental design. The analysis indicated hsa-miR-222 as a stable endogenous reference in relation to other tested candidates, which can be considered a normalizer for the samples of this study under the tested conditions.

### 2.5 Statistical analysis

Data were processed and analyzed using SPSS Statistics version 23 (International Business Machines, New York, United States) (RRID:SCR_016479) and MedCalc version 20.214 (MedCalc Software Ltd., Ostend, Belgium) (RRID:SCR_015044). A normality test, Shapiro-Wilk, was carried out in all quantitative variables. For sociodemographic, clinical-pathological characteristics, and risk factors, categorical data were summarized as absolute and relative frequency, quantitative data were presented as median with interquartile range. Fisher’s Exact Test was performed to determine the association between molecular subtypes with sociodemographic, clinical-pathological characteristics, and risk factors. The Kruskal-Wallis H Test was performed to compare the distributions of quantitative variables according to the molecular subtypes.

The results of the miRNAs were expressed in 2^−ΔΔCt^. To compare the relative expression levels of miRNA between patients with breast cancer and the healthy control group, Mann–Whitney U test was used. ROC curves were generated to assess the ability of miRNAs to differentiate between breast cancer patients and healthy controls. A correlogram was generated using Spearman’s rank correlation coefficient measuring the correlation rank between miRNAs. The adopted level of significance was 5% (*p* < 0.05).

### 2.6 *In-silico* analyses

The miRNAs that showed differential expression with statistical significance were submitted to bioinformatics analysis on online platforms for *in silico* prediction of the main biological pathways involved using mirPath v.3, and the target genes of these miRNAs using miRNet 2.0.

The heat map of biological pathways was generated using a free online bioinformatics tool: DIANA software—mirPath v.3 (DIANA LAB - University of Thessaly, Volos, Greece) (http://www.microrna.gr/miRPathv3) (RRID:SCR_017354) a platform dedicated to the evaluation of miRNA regulatory functions and identification of pathways (Vlachos et al., 2015). DIANA-miRPath v3.0 database and functionality include TargetScan, Tarbase, Kyoto Encyclopedia of Genes and Genomes (KEGG), and Gene Ontology. In the analysis using the Gene Ontology database, the *p*-value limit was set to 1e-20 to eliminate the output of non-relevant data and consequently reduce the analysis noise.

The network diagram of predicted target genes was generated using miRNet 2.0 online software (XiaLab—McGill University, Quebec, Canada) (https://www.mirnet.ca/) (RRID:SCR_024567) which predicts possible genetic targets. This free online tool allows the creation of miRNA-centric multiplex networks integrating key players involved in gene regulation, as well as other molecules of interest from user data. The platform uses the MiRBase, miRTarBase, TarBase, and KEGG databases (Chang et al., 2020). In this analysis, only genes that had at least links with two different miRNAs and genes that are involved in breast cancer pathways were filtered. For graphical representation, an interaction network map between miRNAs and target genes was generated on the same platform.

## 3 Results

### 3.1 Characteristics of the study population

In total, we evaluated 49 patients with breast cancer and 44 healthy women who were part of the control group. Sociodemographic, clinical-pathological characteristics and risk factors of patients with breast cancer were evaluated according to molecular subtypes (Table 1). In the immunohistochemical profile, a predominance of luminal tumors was observed (65.3% of all cases), with 15 patients being classified as Luminal A (30.6%), 11 patients classified as Luminal B (22.4%), and six patients as Luminal HER2 (12.2%). Regarding the non-luminal HER2 subtype, nine patients were identified (18.3%) and from the triple negative subtype, eight patients were identified (16.3%).

**TABLE 1:** Sociodemographic, clinical-pathological characteristics and risk factors according to molecular breast cancer subtypes.

Characteristics	Molecular subtypes						
	All cases (*N* = 49)	Luminal A (*n* = 15)	Luminal B (*n* = 11)	Luminal HER2 (*n* = 6)	Non-luminal HER2 (*n* = 9)	Triple negative (*n* = 8)	*p*-value^†^
Sociodemographic							
Age at diagnosis, years	55 (46.5–62.5)	55 (47-62)	60 (44-77)	44.5 (34.3–51.8)	51 (47-61)	59.5 (50.3–67.5)	0.146
Age group							
< 50 years	17 (34.7)	4 (26.7)	4 (36.4)	5 (83.3)	3 (33.3)	1 (12.5)	0.094
≥ 50 years	32 (65.3)	11 (73.3)	7 (63.6)	1 (16.7)	6 (66.7)	7 (87.5)	
Race/Ethnicity							
White	14 (28.6)	8 (53.3)	2 (18.2)	0 (0.0)	2 (22.2)	2 (25.0)	0.135
BIPOC	35 (71.4)	7 (46.7)	9 (81.8)	6 (100.0)	7 (77.8)	6 (75.0)	
Education level							
Illiterate	5 (10.2)	0 (0.0)	3 (27.3)	1 (16.7)	0 (0.0)	1 (12.5)	
Completed first degree	11 (22.4)	4 (26.7)	3 (27.3)	1 (16.7)	2 (22.2)	1 (12.5)	0.708
Completed second degree	19 (38.8)	5 (33.3)	4 (36.4)	2 (33.3)	4 (44.4)	4 (50.0)	
Completed superior degree or higher	14 (28.6)	6 (40.0)	1 (9.1)	2 (33.3)	3 (33.3)	2 (25.0)	
Family income							
Up to 2 MW	25 (51.0)	5 (33.3)	8 (72.7)	4 (66.7)	5 (55.6)	3 (37.5)	
2 to 4 MW	13 (26.5)	3 (20.0)	3 (27.3)	1 (16.7)	3 (33.3)	3 (37.5)	0.291
5 to 10 MW	7 (14.3)	5 (33.3)	0 (0.0)	0 (0.0)	0 (0.0)	2 (25.0)	
> 10 MW	4 (8.2)	2 (13.3)	0 (0.0)	1 (16.7)	1 (11.1)	0 (0.0)	
Health insurance							
No	38 (77.6)	8 (53.3)	10 (90.9)	5 (83.3)	8 (88.9)	7 (87.5)	0.170
Yes	11 (22.4)	7 (46.7)	1 (9.1)	1 (16.7)	1 (11.1)	1 (12.5)	
Risk factors menopause							
Premenopausal	20 (40.8)	6 (40.0)	5 (45.5)	5 (83.3)	3 (33.3)	1 (12.5)	0.125
Postmenopausal	29 (59.2)	9 (60.0)	6 (54.5)	1 (16.7)	6 (66.7)	7 (87.5)	
Parity							
Nulliparous	4 (8.2)	0 (0.0)	2 (18.2)	1 (16.7)	0 (0.0)	1 (12.5)	0.214
Primiparous/multiparous	45 (91.8)	15 (100.0)	9 (81.8)	5 (83.3)	9 (100.0)	7 (87.5)	
Lactation							
Six months or more	31 (64.8)	6 (40.0)	7 (63.6)	4 (66.7)	8 (88.9)	6 (75.0)	
Less than 6 months or non-brestfeeding	18 (35.2)	9 (60.0)	4 (36.4)	2 (33.3)	1 (11.1)	2 (25.0)	0.174
Family history (1st degree)							
No	34 (69.4)	12 (80.0)	7 (63.6)	6 (100.0)	4 (44.4)	5 (62.5)	0.180
Yes	15 (30.6)	3 (20.0)	4 (36.4)	0 (0.0)	5 (55.6)	3 (37.5)	
Clinical-laboratory laterality							
Right	28 (57.1)	8 (53.3)	5 (45.5)	5 (83.3)	5 (55.6)	5 (62.5)	
Left	19 (38.8)	6 (40.0)	5 (45.5)	1 (16.7)	4 (44.4)	3 (37.5)	0.935
Bilateral	2 (4.1)	1 (6.7)	1 (9.1)	0 (0.0)	0 (0.0)	(0.0)	
Clinical staging							
0—IIA	23 (46.9)	8 (53.3)	3 (27.3)	2 (33.3)	6 (66.7)	4 (50.0)	0.456
IIB—IV	26 (53.1)	7 (46.7)	8 (72.7)	4 (66.7)	3 (33.3)	4 (50.0)	
Histopathological profile							
DCIS	46 (93.9)	13 (86.7)	10 (90.9)	6 (100.0)	9 (100.0)	8 (100.0)	0.664
Lobular	2 (4.1)	2 (13.3)	0 (0.0)	0 (0.0)	0 (0.0)	0 (0.0)	
Mucinous	1 (2.0)	0 (0.0)	1 (9.1)	0 (0.0)	0 (0.0)	0 (0.0)	
Histologic grade							
Well differentiated	4 (8.2)	3 (20.0)	0 (0.0)	0 (0.0)	0 (0.0)	1 (12.5)	
Moderately differentiated	38 (77.6)	12 (80.0)	8 (72.7)	5 (83.3)	7 (77.8)	6 (75.0)	0.319
Poorly differentiated	7 (14.3)	0 (0.0)	3 (27.3)	1 (16.7)	2 (22.2)	1 (12.5)	
Invasion							
No	42 (85.7)	13 (86.7)	9 (81.8)	5 (83.3)	7 (77.8)	8 (100.0)	0.781
Yes	7 (14.3)	2 (13.3)	2 (18.2)	1 (16.7)	2 (22.2)	0 (0.0)	
Lymph node status							
Negative	30 (61.2)	7 (46.7)	3 (27.3)	5 (83.3)	8 (88.9)	7 (87.5)	0.012*
Positive	19 (38.8)	8 (53.3)	8 (72.7)	1 (16.7)	1 (11.1)	1 (12.5)	
MicroRNAs							
miR-210 (*N* = 45)	0.58 (0.22–1.46)	0.30 (0.08–1.22)	1.24 (0.12–1.48)	0.48 (0.24–3.21)	0.80 (0.26–1.94)	0.44 (0.23–0.77)	0.643
miR-195 (*N*= 45)	0.19 (0.03–0.96)	0.15 (0.02–0.68)	0.64 (0.04–2.32)	1.20 (0.13–1.91)	0.72 (0.08–0.78)	0.02 (0.02–1.40)	0.642
miR-34a (*N* = 40)	0.52 (0.03–0.96)	0.42 (0.30–0.73)	0.53 (0.17–2.26)	0.85 (0.28–2.30)	3.29 (0.59–4.57)	0.40 (0.20–2.46)	0.387
miR-16 (*N* = 38)	0.12 (0.02–0.74)	0.22 (0.01–0.67)	0.20 (0.02–0.75)	1.27 (0.17–2.18)	0.37 (0.04–0.78)	0.02 (0.01–0.53)	0.757
miR-21 (*N* = 37)	0.08 (0.01–0.75)	0.16 (0.02–0.92)	0.06 (0.01–0.37)	0.99 (0.05–0.99)	0.52 (0.19–1.15)	0.02 (0.01–0.94)	0.534
miR-10b (*N* = 28)	0.31 (0.08–1.55)	0.14 (0.04–1.98)	0.15 (0.09–0.77)	1.14 (0.33–1.14)	0.28 (0.15–1.62)	0.36 (0.10–2.13)	0.789
miR-1 (*N* = 24)	0.24 (0.03–1.40)	0.39 (0.03–1.40)	0.69 (0.08–2.30)	0.25 (0.01–0.25)	0.19 (0.06–1.79)	0.02 (0.01–0.02)	0.625

Quantitative data presented as median with interquartile range in parentheses. Categorical data summarized as absolute frequency with percentage in parentheses. MW, minimum wage; DCIS, ductal carcinoma in situ; BIPOC, black, indigenous and person of color; HER2, human epidermal growth factor receptor-2.

^†^Kruskal-Wallis H test or Fisher’s exact test; *: *p* < 0.05.

The bold values indicate *p* < 0.05.

The median age of patients was 55 (46.5–62.5) years. Ranging between 32 years and 89 years. Most patients were 50 years old or older (65.3%), and 24.3% were between 50 and 59 years old. BIPOC women were predominant (71.4%). Non-white patients predominated in most molecular subtypes, apart from Luminal A, where a higher number of white women was observed (53.3%). Most patients had completed a second degree (38.8%). Patients were predominantly with a family income of up to two minimum wages (51%), and only 22.4% were attended by private health insurance.

Among risk factors, 29 patients were postmenopausal (59.2%), especially in Luminal A (60.0%), non-luminal HER2 (66.7%), and triple-negative (87.5%) subtypes. Only four women were nulliparous (8.2%). 31 women (64.8%) reported that they breastfed for more than 6 months. On first-degree relative that presents neoplasm linked to the heredity of breast cancer, specifically breast, ovarian, colorectal, endometrial, central nervous system, and/or pancreatic cancer; 15 patients (30.6%) reported the presence of neoplasia on relatives.

Regarding the clinical-laboratory characteristics, in 28 patients the tumor was located in the right breast (57.1%) and 19 patients had a tumor located on the left side (38.8%). There was a predominance of late diagnosis (53.1%), especially in Luminal B patients (72.7%). The predominant histopathological type was Ductal Carcinoma *in Situ* (DCIS) (93.9%). The predominant histological grade was moderately differentiated (77.6%) and it was predominant in all molecular subtypes ranging from 72.7% to 83.3% of cases. The well-differentiated grade was present only in Luminal A tumors and in a single triple-negative case. Vasculoneural invasion was not found in most cases (85.7%). Only seven patients (14.3%) had vascular and/or perineural invasion present. In all characteristics mentioned above, there were no statistically significant differences in their distribution according to molecular subtypes (*p* > 0.05). Only in lymph node status were there statistically significant differences (*p* = 0.012). Lymph nodes free of metastatic cells were found in 61.2% of all cases. However, lymph node involvement was observed in most Luminal A (53.3%) and Luminal B (72.7%) tumors.

### 3.2 miRNA expression in plasma samples

Plasma levels of miR-210, miR-195, miR-34a, miR-16, miR-21, miR-10b, and miR-1 were measured and compared in patients with breast cancer and healthy controls. The relative expression of the miRNAs was expressed in 2^−ΔΔCT^.

**FIGURE 1:**
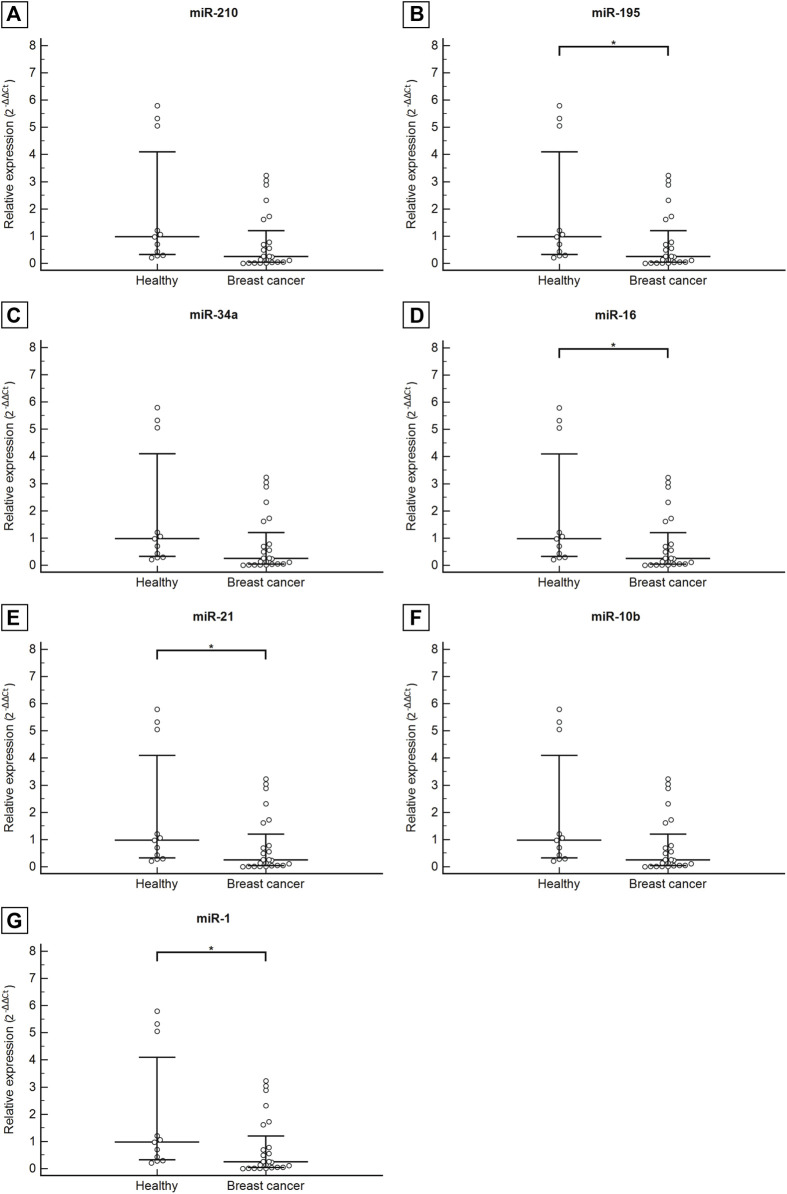
Circulating levels of miR-210 **(A)**, miR-195 **(B)**, miR-34a **(C)**, miR-16 **(D)**, miR-21 **(E)**, miR-10b **(F)**, miR-1 **(G)** in healthy control group compared with patients with breast cancer. Data are graphically represented as median and interquartile range (IQR). Abbreviation: *p < 0.05.

The results demonstrate that all miRNAs analyzed in the plasma of patients with breast cancer were down-expressed in relation to the healthy control group, with a 0.65 mean fold decrease for miR-210, 0.16 for miR-195, 0.32 for miR-34a, 0.09 for miR-16, 0.08 for miR-21, 0.17 for miR-10b and 0.25 mean fold decrease for miR-1. Statistically significant differences in miR-195, miR-16, miR-21, and miR-1 expression levels in breast cancer patients in comparison to healthy controls were recorded. For miR-195, the median circulating levels were 0.19 (0.03–0.96) for the breast cancer patient group and 1.18 (0.18–2.10) for the healthy control (*p* = 0.008). The same was observed in the analysis of circulating levels of miR-16 with a median value of 0.12 (0.02–0.74) for patients with breast cancer and 1.23 (0.34–2.51) for healthy control (*p* = 0.004); miR-21 with values of 0.08 (0.01–0.75) for breast cancer patients and 0.94 (0.38–2.48) for healthy control (*p* <0.0001); and lastly of miR-1 with values of 0.24 (0.03–1.40) for breast cancer patients and 0.97 (0.29–5.04) for healthy control (*p* = 0.022). The median circulating levels of miR-210, miR-34a, and miR-10b in breast cancer patients were 0.58 (0.22–1.46), 0.52 (0.03–0.96), and 0.31 (0.08–1.55), respectively. As well, the levels of the healthy control group were 0.88 (0.30–1.94), 1.62 (0.39–2.57), and 1.86 (0.12–3.14), respectively. No statistical significance was observed in the expressions of these miRNAs between the comparison groups (Figure 1).

The study also aimed to evaluate the association between plasma levels of miRNAs and the immunohistochemical profile of patients with breast cancer. All miRNAs did not show statistically significant differences between groups. These data did not show any statistical significance. The sample size may have limited a more robust statistical analysis (Table 1).

### 3.3 Potential diagnostic value of multiple miRNAs in plasma

**FIGURE 2:**
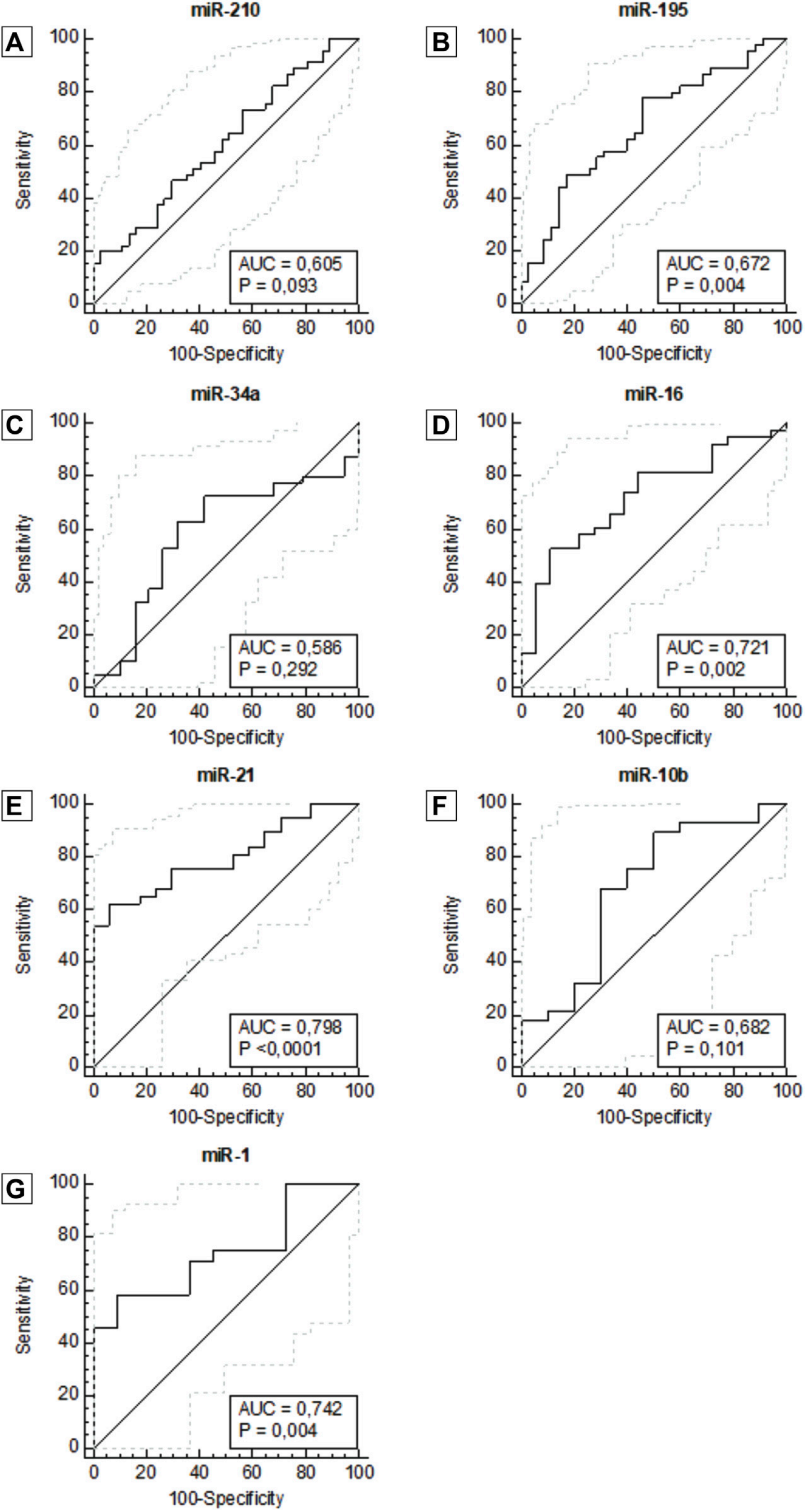
ROC curve for miR-210 **(A)**, miR-195 **(B)**, miR-34a **(C)**, miR-16 **(D)**, miR-21 **(E)**, miR-10b **(F)**, miR -1 **(G)**, according to diagnosis. Abbreviation: AUC: Area under the ROC curve.

The data were then utilized to generate ROC curves and calculate the area under the ROC curve to determine the potential of each plasma miRNA as a diagnostic biomarker to differentiate patients with breast cancer patients from healthy women. ROC curve analysis of miR-21 (AUC = 0.798, 95% CI: 0.682–0.914, *p* <0.0001), miR-1 (AUC = 0.742, 95% CI: 0.576–0.909, *p* = 0.004), miR-16 (AUC = 0.721, 95% CI: 0.581–0.861, *p* = 0.002) and miR-195 (AUC = 0.672, 95% CI: 0.553–0.792, *p* = 0.004) showed better diagnostic accuracy, with preeminent AUC in descending order showing statistically significant (Figure 2; Table 2).

**TABLE 2:** Diagnostic performance of miRNAs using ROC and AUC curve analysis in breast cancer patients.

microRNA	AUC (95 CI %)	Sensitivity (%)	Specificity (%)
miR-210	0.605 (0.482–0.728)	20.0	97.3
miR-195	0.672 (0.553–0.792)*	77.8	54.3
miR-34a	0.586 (0.427–0.744)	62.5	68.4
miR-16	0.721 (0.581–0.861)*	52.6	88.9
miR-21	0.798 (0.682–0.914)**	62.2	94.1
miR-10b	0.682 (0.464–0.900)	89.3	50.0
miR-1	0.742 (0.576–0.909)*	58.3	90.9

ROC, receiver operating characteristic; AUC, area under the ROC; 95 CI%, confidence interval at the level 95%.

*p <0,05; **p <0,0001.

**FIGURE 3:**
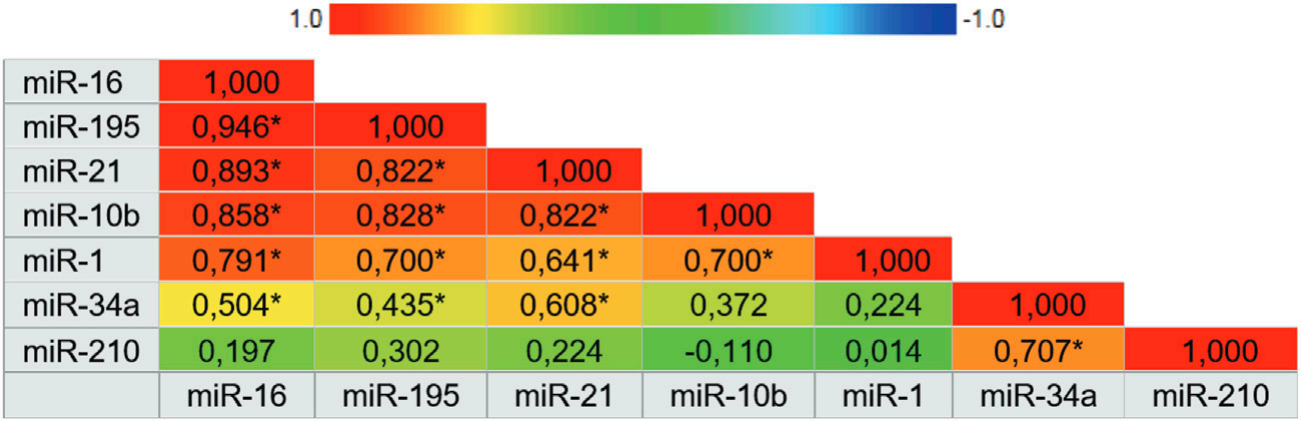
Correlogram representing the expression correlation matrix of the seven microRNAs. The color of the cells varies according to the magnitude of correlation, ranging from dark red for positive correlations to dark blue for negative correlations. Abbreviation: * Correlation is significant at 0.01 level.

miR-210, miR-21, miR-1, and mir-16 showed the highest specificities values with results of 97.3%, 94.1%, 90.9% and 88.9%, respectively. Following, miR-10b and miR-195 showed the highest sensitivity values of 89.3% and 77.8%, respectively. There was not a single isolated marker with high sensitivity and high specificity combined. Our results demonstrate that the expression of miR-1, miR-16, miR-21, and miR-195 in plasma samples is accurate enough to distinguish women with breast cancer from healthy women. In the same way that miR-210, due to its high specificity, has an important role in the next analyses. To complement these data, we also constructed a correlogram that demonstrated a very strong/strong, positive, and significant correlation between miRNAs miR-195, miR-21, miR-16, and miR-10b (Figure 3).

**FIGURE 4:**
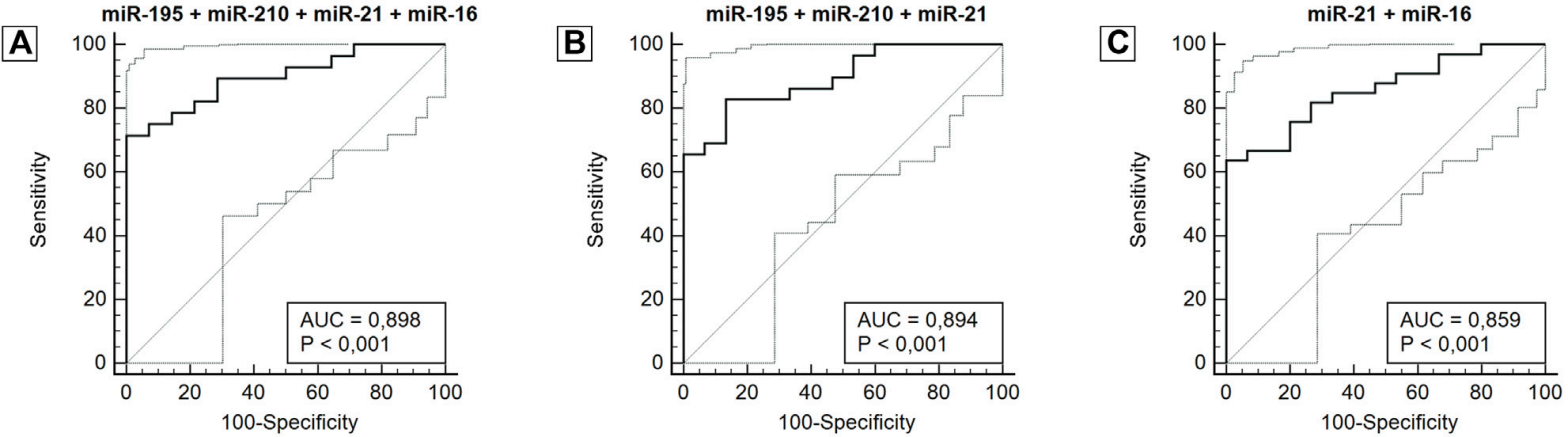
ROC curve for miRNA panels miR-195 + miR-210 + miR-21 + miR-16 **(A)**, miR-195 + miR-210 + miR-21 **(B)**, miR-21 + miR-16 **(C)**, according to diagnosis. Abbreviation: AUC: Area under the ROC curve.

After analyzing the ROC curve of miRNAs, the combination of miRNAs with the most relevant and potential results was performed to evaluate the performance of miRNA panels. Thus, miR-34a, miR-10b, and miR-1 were excluded from further investigations. Several combinations were performed, three of which stood out in combination with a panel of four miRNAs (miR-195 + miR-210 + miR-21 + miR-16), three miRNAs (miR-195 + miR-210 + miR-21) and two miRNAs (miR-21 + miR-16). The panel with four miRNAs had an AUC of 0.898 (0.765–0.970), a sensitivity of 71.4%, and a specificity of 100.0%. The panel with three miRNAs had an AUC of 0.894 (0.764–0.967), a sensitivity of 82.8%, and a specificity of 86.7%. And lastly, the panel with two miRNAs showed an AUC of 0.859 (0.728–0.942), a sensitivity of 63.3%, and a specificity of 100.0%. The miRNA combination in panels significantly improves the results (Figure 4; Table 3).

**TABLE 3:** Diagnostic performance of a combined miRNAs panel using ROC and AUC curve analysis in breast cancer patients.

microRNA	AUC (95 CI %)	Sensitivity (%)	Specificity (%)
miR-195 + miR-210 + miR-21 + miR-16	0.898 (0.765–0.970)**	71.4	100.0
miR-195 + miR-210 + miR-21	0.894 (0.764–0.967)**	82.8	86.7
miR-210 + miR-21+ miR-16	0.879 (0.745–0.957)**	70.0	92.9
miR-195 + miR-21+ miR-16	0.867 (0.734–0.949)**	67.7	93.3
miR-21 + miR-16	0.859 (0.728–0.942)**	63.3	100.0
miR-195 + miR-21	0.857 (0.726–0.941)**	59.4	100.0
miR-210 + miR-21	0.826 (0.691–0.919)**	78.8	81.2
miR-195 + miR-210 + miR-16	0.825 (0.687–0.921)**	80.0	76.5
miR-210 + miR-16	0.809 (0.671–0.907)**	65.6	88.2
miR-195 + miR-16	0.757 (0.618–0.865)*	64.7	83.3
miR-195 + miR-210	0.712 (0.592–0.813)*	74.4	65.6

ROC, receiver operating characteristic; AUC, area under the ROC; 95 CI%, confidence interval at the level 95%.

*p <0.05; **p <0.0001.

### 3.4 Bioinformatics analysis of miRNAs

**FIGURE 5:**
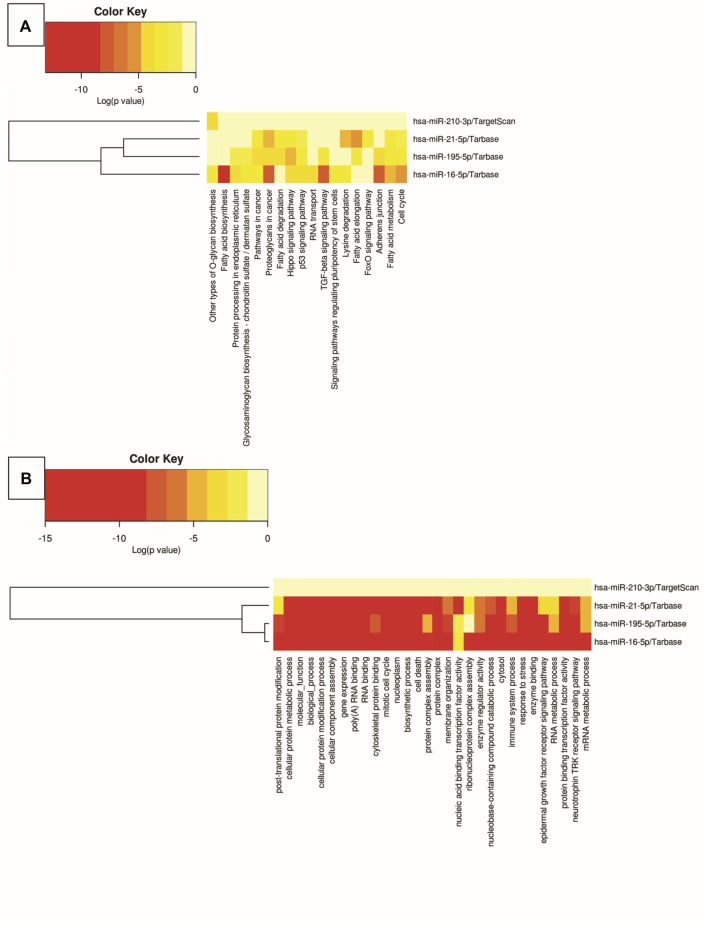
Heat map of biological pathway analysis using Kyoto Encyclopedia of Genes and Genomes **(A)** and Gene Ontology **(B)**. The color of the cells varies according to *p*-value, expressed in log10, ranging from yellow to red.

*In silico* analyses were carried out to demonstrate the interactions between the miRNAs miR-210, miR-195, miR-21, and miR-16 and biological pathways, using DIANA software - mirPath v.3. The results were presented through heat maps (Figure 5). Several cancer-related pathways have been identified, such as: p53 gene signaling pathways, glycosaminoglycan biosynthesis, TGF-β signaling pathway, signaling pathways that regulate pluripotency of stem cells, FoxO signaling pathway, cell death, response to stress, RNA metabolic processes and assembly of cellular components that may involve morphogenesis, intracellular transport, and catabolic processes.

**FIGURE 6:**
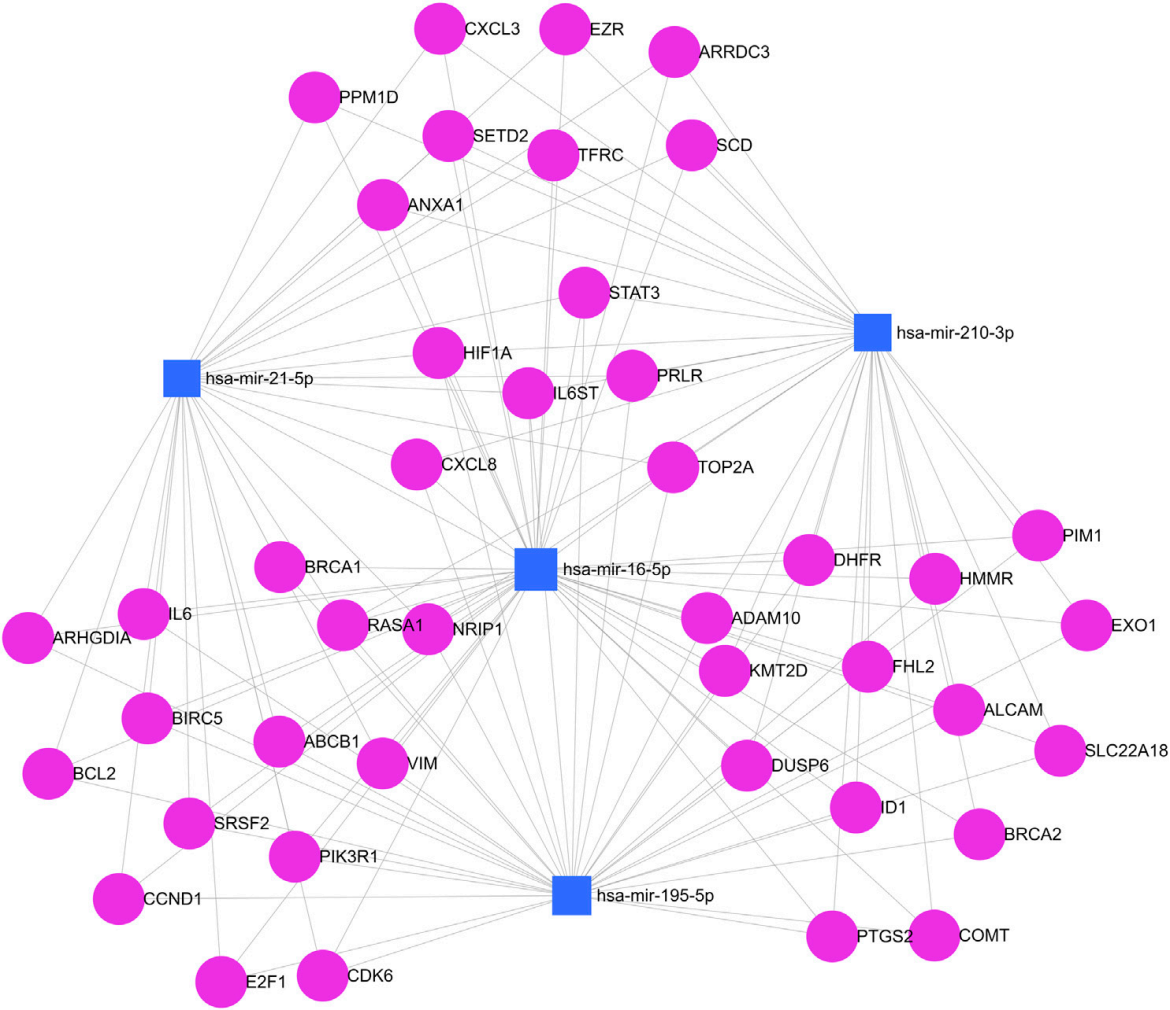
Simultaneous analysis of predicted target genes for miRNAs. Blue squares represent miR-210, miR-195, miR-21, and miR-16. Pink circles represent target genes. Each gray line represents a link between a gene and a miRNA in this network map.

Finally, the computational prediction of target genes that are involved in breast cancer pathways and that have bindings with at least two different miRNAs was evaluated using miRNet 2.0 software. In total, 42 genes were identified under these conditions. Figure 6 shows a network map between analyzed miRNAs and regulated target genes. The graph shows interactions between miR-16, miR-21, miR-195, and miR-210 with different target genes of breast cancer pathways at the same level, including interactions with highly penetrant genes such as BRCA1 and BRCA2. The interaction is preeminent between miR-16 and miR-195 which is associated with correlation data described earlier.

### 3.5 Discussion

The data show all seven miRNAs analyzed of patients with breast cancer were underexpressed in relation to the healthy control group. However, only miR-195, miR-16, miR-21, and miR-1 have statistically significant differences between these two groups. All miRNAs did not show statistically significant differences between the immunohistochemical profile. ROC curve analysis of miR-21, miR-1, miR-16, and miR-195 showed better diagnostic accuracy. miR-210, miR-21, miR-1 and mir-16 showed the highest specificities values as well miR-10b and miR-195 showed the highest sensitivity values. The miRNA combination in panels significantly improves their results. *In silico* analysis demonstrated interactions between miRNAs and several cancer-related pathways together with a prediction of target genes that are involved in these pathways. Next, we will discuss the role of miRNAs evaluated in clinical practice and their relationship with biological pathways.

### 3.6 MiRNAs in clinical practice: assessing their role and connection with biological pathways

#### 3.6.1 MicroRNA-210 (miR-210)

miR-210 is down-expressed in relation to the healthy control group. However, miR-210 perform an oncogene role in breast cancer, it is normally overexpressed both in tissue samples and in serum or plasma, and is associated with a worse prognosis (Evangelista et al., 2021). Our underexpression findings diverge from what is normally detected. It is found in underexpression in bladder cancer, angiosarcoma, and glioblastoma. Although, it is upregulated in breast cancer (Khalilian et al., 2023), There is also an increase in expression levels of miR-210 and BRCA1 mRNA in TNBC patients after treatment and with a worse prognosis (Boukerroucha et al., 2015). miR-210 participates in the proliferation of breast cancer cells, particularly triple-negative cells, and is related to lymph node metastases through targets such as CCND1 and RUNX3 (Khalilian et al., 2023; Santana et al., 2023). *In vivo* results show that a hypoxic microenvironment induces an upregulation of miR-210 leading to increased breast cancer progression by reducing the expression of E-cadherin protein in breast cancer stem cells, whereas the inhibition of this miRNA suppresses cell proliferation and consequent metastasis (Tang et al., 2018). Thus, its characteristics and biological interactions make it a potential biomarker for diagnosis, prognosis, and clinical staging (Evangelista et al., 2021; Khalilian et al., 2023; Santana et al., 2023). Despite the inconstancy found in miR-210, the findings for all other miRNAs are consistent with a wide variety of studies.

#### 3.6.2 MicroRNA-195 (miR-195)

For miR-195, it has a role as a tumor suppressor in several types of cancer, including breast cancer. Its overexpression can suppress tumor cell invasion, and cell cycle progression and induce apoptosis (Yang et al., 2013). miR-195 targets *de novo* lipogenesis genes, a hallmark in cancer cells especially in aggressive tumors, and that are related to the production of cell membranes for rapid cell proliferation through action on overexpressed target genes in breast cancer such as BCL-2, FASN, ACACA, and HMGCR (Singh et al., 2015; Chen et al., 2022). miR-195 also regulates mitochondrial function from MFN2 and consequently affecting mitochondrial morphology and inducing defects in mitochondrial respiration from changes in oxygen consumption rates (Purohit et al., 2019). Several studies have evaluated the relative expression levels of miR-195 with treatment responses. miR-195 regulates chemoresponse via SEMA6D in which reduced levels of SEMA6D were significantly associated with increased chemoresistance (Baxter et al., 2021). Rizzo et al. (2017) evaluated the response to doxorubicin and demonstrates that miR-195 upregulation is associated with increased response to treatment and this miRNA is involved in pathways related to drug sensitivity/resistance as previously described by Yang et al. (2013). Zhao et al. (2014) evaluating 210 patients with breast cancer, identified that miR-195 was underexpressed in the serum of these patients. The sensitivity and specificity of miR-195 in diagnosis were 69.0% and 89.2%. Additionally, McAnena et al. (2019) demonstrate that miR-195 plasma levels are significantly decreased in metastatic breast cancer patients compared to locally confined Luminal A breast cancer patients and healthy controls. A panel with miR-195 and miR-331 achieved an AUC of 0.902 for distinguishing metastatic from local breast. miR-195 has been shown to have greater sensitivity than the cited studies. We emphasize that the lowest median levels of relative expression of miR-195 were present in the triple-negative subtype, which is the most aggressive subtype.

#### 3.6.3 MicroRNA-34a (miR-34a)

miR-34a transcription is under the control of p53 and acts as a tumor suppressor gene inducing cell cycle arrest in G1, senescence, and apoptosis, additionally to suppressing proliferation and tissue invasion by inhibiting BCL-2 and SIRT1 (Misso et al., 2014). It also has other molecular targets such as MYC, CDK6, and MET. In tissue samples, its upregulation is associated with an unfavorable tumor phenotype being associated with positive lymph node status, late staging, HER2 positivity, and high proliferation rates (Peurala et al., 2011). miR-34a negatively regulates Notch1 expression, thus becoming a regulatory factor involved in tumor invasion and metastasis (Rui et al., 2018). It is also involved in immune response and carcinogenesis through IL-6 activation. This IL-6 signaling pathway positively interacts with the Stat3 pathway, resulting in tumor promotion and an increase in breast cancer stem cells, mainly in the triple-negative subtype (Weng et al., 2019). The reduction of its expression is associated with tumor progression and a worse prognosis. Even downregulated, the highest levels of miR-34a in our study were measured in non-luminal HER2 and luminal HER2 molecular subtypes. Shaban et al. (2022) evaluated this miRNA as a response to treatment. miR-34a was downregulated in the pre-treatment phase and after chemo-radiotherapy miR-34a levels increased significantly, proving to be a promising biomarker in the evaluation of response to treatment. Zaleski et al. (2018) identified low expression of miR-34a in patients with breast cancer evaluated in Germany. The diagnostic accuracy and sensitivity increased when combining miR-34a with CEA and CA 15-3 markers, in addition, to assisting both in the differential diagnosis and cancer staging. We did not use miR-34a in combinations with other microRNAs because it had low accuracy and non-significant results. A similar situation occurred with Chen et al. when assessing a panel of miRNAs in 260 patients with Breast Invasive Ductal Carcinoma. After the validation cohort, miR-34a was excluded from the study, as its value lacked of statistical significance (Chen et al., 2021).

#### 3.6.4 MicroRNA-16 (miR-16)

miR-16 plays a role in controlling various cellular functions and biological pathways such as apoptosis, cell differentiation, and cell proliferation (Cai et al., 2018). It is also associated with the BRCA1 and BRCA2 genes, being upregulated by them (Tommasi et al., 2021). Specifically, in breast cancer, miR-16 inhibits PGK1 expression and consequently inhibits aerobic glycolysis, a hallmark of cancer, decreasing glucose uptake as well as lactate and ATP production. With the decrease in lactate production, the extracellular acidification rate also decreases. PGK1 suppression by miR-16 leads to suppression of tumor growth and metastasis as aerobic glycolysis is a key point in cell proliferation, migration, and tissue invasion (Ye et al., 2020). Other targets have already been described, such as ANLN, which inhibits proliferation, cell migration, and tissue invasion, in addition to affecting the cell cycle, inducing apoptosis of breast tumor cells and arresting cells in the G2/M phase (Wang et al., 2021). Qu et al. (2017); Wang et al. (2021) evaluating breast cancer cells and tissues identified that miR-16 had significantly lower expression levels in tumor tissue compared to epithelial breast cells and normal tissues. However, Enders et al. found significantly elevated levels of miR-16 in the plasma of breast cancer patients compared to healthy controls (Ng et al., 2013). Our results corroborate with Qu et al. and Wang et al. in which miR-16 was underexpressed and showed significant differences between breast cancer patients compared to the healthy control group.

#### 3.6.5 MicroRNA-21 (miR-21)

Regarding miR-21, as it is an oncogenic miRNA, we expected it to be overexpressed. However, it was underexpressed, in addition to having the highest AUC and one of the highest specificities of microRNAs evaluated. miR-21 promotes breast cancer progression and metastasis due to its suppression of LZTFL1, which acts on the beta-catenin protein signaling pathway, inactivating the epithelial-mesenchymal transition (Wang et al., 2019). miR-21 also has an inhibitory action on TIMP3, PDCD4, TPM1 and RECK. All these have protein products with known anti-tumorigenic, anti-proliferative, anti-invasive, and anti-angiogenic actions. Li et al. (2016) performed a meta-analysis of six studies with 438 patients and 228 healthy controls and demonstrated that miR-21 can be an accurate biomarker for early diagnosis and prognosis, being implicated in epithelial-mesenchymal transition, cell migration, and invasion control. miR-21 proves to be a potential biomarker for a treatment follow-up. Khalighfard et al. (2018) identified that plasma levels of miR-21 are reduced after treatment, whether surgical treatment, chemotherapy, or radiotherapy. Regarding diagnosis, current findings in breast cancer are controversial. Wang et al. observed miR-21 was overexpressed in the plasma of women with breast cancer when compared to healthy women or those with benign breast tumors (Wang et al., 2019). However, Wu et al. (2012) analyzed the serum expression of several miRNAs in 88 patients with breast cancer in stages I and II, they observed that miR-21 was hypoexpressed in relation to the healthy control group. Chen et al. when evaluating 90 patients with breast cancer and comparing them to healthy women and patients with benign breast lesions, identified significant differences in levels of miR-21 in the serum of breast cancer patients compared to healthy women. However, it failed to identify differences in quantifications compared to patients with benign breast lesions (Chen et al., 2019). These inconsistencies in studies can be derived from the disparity of samples origin to different analytical methods, different platforms in studies, or the metabolic status of patients that can influence the molecular composition of tumor producing different results (Khalighfard et al., 2018; Meerson et al., 2019).

#### 3.6.6 MicroRNA-10b (miR-10b)

miR-10b is regulated by the Twist transcription factor and upregulates metastasis and tissue invasion by inactivating HOXD10, resulting in an increased expression of the pro-metastatic RHOC gene (Ma et al., 2007). miR-10b also upregulates tissue invasion through modulation of TGF-b1-induced epithelial-mesenchymal transition. miR-10b targets other genes such as PLK1, CCNA2, BUB1, and HOXD4. Inhibition of miR-10b reverses breast cancer cell proliferation. Therefore, its expression may be correlated with aggressiveness in breast cancer (Han et al., 2014; Raval et al., 2022). Despite being identified as upregulated in several types of cancer, several studies show that it may also be underexpressed in breast cancer or without significant differences compared to healthy women, analogous to the results of our study. Zhang et al. (2018) identified that there is no difference in the expression of miR-10b in tissue samples between different molecular subtypes of breast cancer, although miR-10b is related to clinical stage, overall survival, positive lymph node status, high Ki-67, and tumor size, thus being considered a prognostic marker. Nassar et al. (2014) evaluating breast tissue from 57 patients with breast cancer in Lebanon from a miRNA panel, identified downregulated miR-10b when compared with normal breast tissue, with a AUC of 0.502. The most prominent differences were in ER and PR negative patients. Its expression is also correlated with body mass index, in which miR-10b expression levels are reduced the higher patient’s body mass index is (Meerson et al., 2019).

#### 3.6.7 MicroRNA-1 (miR-1)

miR-1 acts as a tumor suppressor, promoting the inhibition of tumor growth and acting with multiple target genes, such as CDK4, TMSB4X, WASF2, TWF1, CNN3, and CORO1C which are genes involved in cell cycle and metastasis. miR-1 causes cell cycle arrest at the G1/G0 phase, resulting in inhibited breast cancer cell proliferation (Liu et al., 2017). Overexpression of miR-1 in breast cancer decreases cell proliferation, and invasion and induces apoptosis *in vitro* and *in vivo* through reduced Bcl-2 expression (Peng et al., 2020). miR-1 can also alter EVI-1 expression in breast cancer cells, consequently inhibiting cell proliferation, inducing apoptosis, and modulating epithelial–mesenchymal transition (Wu, 2018). (Wu, 2018); Peng et al. (2020); Chen et al. (2021) identified that miR-1 was shown to be significantly downregulated in breast cancer tissue and serum compared with healthy controls. miR-1 can also be used as a biomarker of cardiotoxicity induced by chemotherapy drugs used in breast cancer, such as doxorubicin and anthracycline (Rigaud et al., 2017; Pereira et al., 2020).

### 3.7 Improving diagnostic accuracy: application of miRNAs panel

The combination of miRNA in panels has been widely used in studies to increase diagnostic accuracy. McDermott et al. evaluated 54 patients with Luminal A breast cancer against healthy controls and identified 76 differentially expressed miRNAs. From a combination of miR-29a, miR-181a, and miR-652, an AUC of 0.800 was obtained, with a sensitivity of 77% and specificity of 74% in discrimination of breast cancer in comparison with the healthy control group (Mcdermott et al., 2014). Zhang et al. (2015) conducted a study with 76 patients with breast cancer evaluating a panel of 96 miRNAs, of which 23 miRNAs were significantly downregulated in patients with breast cancer compared to healthy controls. A three miRNA panel (miR-199a, miR-29c, and miR-424) showed the highest diagnostic accuracy (AUC = 0.888, 95% CI 0.781–0.995) to distinguish breast cancer patients from healthy subjects and successfully confirmed in the validation set with AUC of 0.901 (95% CI 0.850–0.951), the sensitivity of 77.6% and specificity of 84.6%. Enders et al. evaluated 260 patients with breast cancer in a series of stages of discovery, selection, training, and validation of diagnostic biomarkers. From the combination of miR-145 and miR-451, in the blind validation, an AUC of 0.931 was obtained (95% CI 0.886–0.977) with a sensitivity of 83% and specificity of 93% in the discrimination of breast cancer in comparison with healthy women (Ng et al., 2013). The combination of four microRNAs (miR-195 + miR-210 + miR-21 + miR-16) obtained an AUC of 0.898 (95% CI 0.765–0.970) at a sensitivity of 71.4%, and a specificity of 100.0%. A result significantly superior to McDermott et al., but equivalent to Zhang et al. and lower than Enders et al., in addition to having higher specificity and lower sensitivity than these three studies.

### 3.8 Challenges and limitations in the clinical application of miRNA

Despite the rapid evolution and a vast number of clinical and preclinical studies involving miRNAs as a breast cancer biomarker, there are still several obstacles that must be overcome before miRNA profiles can be routinely incorporated into clinical practice. One reason is the lack of reproducibility in data across different breast cancer studies, leading to inconsistent results. The difference in the expression of miRNAs in different types of samples such as plasma and serum; the use of different protocols for blood sample collection and processing; different study designs; the method used for the extraction of the circulating miRNAs; and the normalization method adopted are the main factors. As well as the relative expression levels of miRNAs depend on various factors that extend beyond breast cancer itself, including lifestyles (such as smoking status, diet, and physical activity) and individual diversity (such as sex, ethnicity, and age), can generate intra and inter-individual variability. And lastly, downregulation or upregulation of the same miRNA can be found in more than one type of cancer; this is not a finding exclusive to breast cancer. The use of a panel of miRNAs helped mitigate this issue, enhancing the accuracy and precision of the results (Gareev et al., 2020; Precazzini et al., 2021). miRNAs have the potential to be a non-invasive biomarker for breast cancer diagnosis as demonstrated by our study, and their validation with a multicenter study of a larger population, and involving blinded samples are necessary to confirm the clinical usefulness of miRNA panels for breast cancer detection.

### 3.9 Current strategies and future prospects in early breast cancer detection

The favorable clinical prognosis of BC is intricately linked to the implementation of advanced and effective strategies for the early diagnosis of the condition. Although mammography remains the fundamental diagnostic approach for the early identification of BC, it has limitations including the occurrence of false negatives, false positives, and unnecessary biopsies (Tabar et al., 2003). Breast ultrasound is used as an auxiliary exam, but it depends on equipment, subjective interpretation and can lead to false positives (Majid et al., 2003). Additionally, other imaging-based approaches, including X-rays, computed tomography, and magnetic resonance imaging, are associated with an increased frequency of highly invasive biopsies to determine the malignant or benign nature of the tumor (Zhang et al., 2022). Furthermore, some lesions can be highly variable with a probability of malignancy ranging from 3% to 95%, causing patients with benign lesions to undergo invasive examinations and, in some cases, surgery as the method of first choice, unnecessarily (Giussani et al., 2021). Invasive biopsies have disadvantages, including discomfort and even trauma for patients and potential side effects (Hirahata et al., 2022).

Thus, the liquid biopsy has emerged as a viable solution to address these limitations and act as a minimally invasive first-choice complementary test enables early detection for BC screening. Moreover, the minimally invasive blood tests increase the acceptance by patient acceptance, while reducing costs, of population-based screening for BC. Consequently, it offers simplicity, is painless and avoids the recovery periods and side effects of tissue biopsy. In this context, circulating miRNAs have emerged as biomarkers for the early detection and diagnosis of BC in liquid biopsies (Sharifi et al., 2022). They can serve as a basis for developing precise diagnostic assays which, combined with existing diagnostic techniques, might significantly enhance the sensitivity and specificity of BC detection. In this setting, our study analyzed circulating microRNAs in search of diagnostic biomarkers capable of discriminating women with BC from healthy women within a population primarily consisting of individuals BIPOC. The data provide valuable complementary information in a population with genetic heterogeneity, which will contribute to the search unique expression patterns of miRNAs could act as reliable “biomolecular signatures” for BC.

Our results could open new perspectives for medical application in breast cancer diagnosis. We were able to identify four miRNAs (miR-210, miR-16, miR-21, and miR-195) with significantly altered results in breast cancer patients compared to healthy controls. The panel with these microRNAs showed high accuracy. This sustains the potential role of a miRNAs panel as non-invasive biomarkers for breast cancer detection. These findings emphasize the potential of miRNAs as a tool molecular highly precise diagnostic assays to the early detection of BC. Upon integration with contemporary diagnostic methodologies, these assays will hold the capability to significantly increase both the sensitivity and specificity. Also, we assessed the discriminatory capacity of these miRNAs across BC molecular subtypes. However, their expression profiles failed to differentiate between various BC immunohistochemical profiles. It is likely that the sample size may have constrained a more comprehensive and robust statistical analysis for this assay. Additional investigations involving diverse populations are imperative to authenticate the prosperous outcomes already attained in employing miRNAs as non-invasive biomarkers for BC.

## 4 Conclusion

In this study, we demonstrated four miRNAs-based signatures able to discriminate women with BC from healthy women within a population primarily consisting of individuals BIPOC. In summary, our results demonstrate that miRNA expression panels can be used as a promising molecular biomarker in the diagnosis of breast cancer. Specifically, we identified that miR-210, miR-16, miR-21, and miR-195 and their combinations can help in the diagnosis of breast cancer accurately displaying good sensitivity and specificity. Although our identified signatures are unlikely to be used alone independently achieve precise BC predictions, our study supports the application the circulating miRNAs as a minimally invasive first-choice complementary test enables early detection for BC screening. This integration holds the promise of enhancing imaging-based screening modalities for early BC diagnosis, potentially mitigating the necessity for unwarranted biopsies in a substantial proportion of women. Subsequent investigations are imperative to validate the analytical performance of our identified signatures and conduct a thorough assessment of their clinical application.

## Data Availability

The original contributions presented in the study are included in the article/Supplementary Material, further inquiries can be directed to the corresponding authors.
